# Circular RNA Expression Profiles in Nasopharyngeal Carcinoma by Sequence Analysis

**DOI:** 10.3389/fonc.2020.00601

**Published:** 2020-04-30

**Authors:** Jing Yang, Yongqian Gong, Qingshan Jiang, Lijun Liu, Shuyan Li, Quanjun Zhou, Fang Huang, Zhifeng Liu

**Affiliations:** ^1^Department of Gastroenterology, The First Affiliated Hospital of University of South China, Hengyang, China; ^2^Department of Otorhinolaryngology, The First Affiliated Hospital of University of South China, Hengyang, China

**Keywords:** circular RNA (circRNA), nasopharyngeal carcinoma (NPC), high-throughput sequencing, biomarkers, microRNA (miRNA)

## Abstract

Circular RNAs (circRNAs), as a burgeoning sort of non-coding RNAs (ncRNAs), can regulate the expression of parental genes as miRNA sponges. This study was designed to explore the circRNA expression profile of nasopharyngeal carcinoma (NPC). High-throughput sequencing was performed to identify the circRNA expression profile of NPC patients compared with healthy controls. A total of 93 upregulated circRNAs and 77 downregulated circRNAs were identified. The expression levels of the top three upregulated and three downregulated circRNAs annotated by circBase were validated by quantitative real-time PCR (qRT-PCR). GO and KEGG analyses showed that these differentially expressed circRNAs were potentially implicated in NPC pathogenesis. CircRNA-miRNA-target gene network analysis revealed a potential mechanism that hsa_circ_0002375 (circKITLG) may be involved in NPC through sponging up miR-3198 and interfering with its downstream targets. Silencing of circKITLG inhibited NPC cell proliferation, migration, and invasion *in vitro*. This study provides a leading and fundamental circRNA expression profile of NPC.

## Introduction

Nasopharyngeal carcinoma (NPC), the most common head and neck cancer, is associated with remarkable distinct geographical distribution and racial differences, and it is highly prevalent in east and southeast Asia (1, 2). The main causes of NPC include Epstein-Barr virus (EBV), genetic susceptibility, environmental factors, and so on (1). The radiotherapy and chemotherapy treatments used to treat NPC have been advanced. However, 30% of NPC patients will develop local relapsed and distant metastasis, and the outcomes of these patients remain frustrating (3, 4). Searching for the underlying NPC novel targets would help facilitate clinical treatment strategies.

CircRNAs are a novel class of ncRNA molecules, which are single-stranded, have covalently joined head 3′ and tail 5′, and are produced via backsplicing (5, 6). Due to the particular covalently closed circular molecular structure, circRNAs are highly resistant to degradation and more stable than conventional linear RNA (7). Though circRNAs were first identified in RNA viruses in the 1980's, they were initially considered to be splicing-associated noise (8). Currently, much research has demonstrated that circRNAs can function as microRNA (miRNA) sponges (9). Based on the function, more and more studies have revealed that circRNAs play an essential role in the regulation of gene expression and in physiology, pathology, and the initiation and progression of human diseases, especially tumorigenesis (10, 11).

Recently, circRNAs have been indicated to be potential biomarkers or therapeutic targets in various cancers, such as colorectal cancer (12), pancreatic cancer (13), hepatocellular carcinoma (14), bladder carcinoma (15), and lung adenocarcinoma (16). Exome capture transcriptome sequencing was used to compile a cancer circRNA landscape across 40 cancer types by Arul M. Chinnaiyan et al. (17). However, circRNA expression profiling and circRNAs as biomarkers in nasopharyngeal carcinoma have not been reported. Here, we profiled potential circRNA biomarkers in nasopharyngeal carcinoma presented by high-throughput sequencing.

## Materials and Methods

### Patients and Tissue Specimen

Four fresh nasopharyngeal carcinoma tissue specimens and four matched healthy tissue specimens were acquired from nasopharyngeal carcinoma and nasopharyngitis patients via biopsy for circRNA high-throughput sequencing. Then, a total of 41 matched specimens, containing the specimens for sequencing, were used to validate the circRNA expression by qRT-PCR. The experimental tissue specimens were diagnosed based on strict pathologic examination. They had no other tumor history and did not undergo radiotherapy and chemotherapy before biopsy. Pathologic and clinical characteristics of nasopharyngeal carcinoma patients were based on the American Joint Committee on Cancer (AJCC) and the Union for International Cancer Control (UICC) TNM classification. Consent documents were obtained from all patients, and the Medical Ethics Committee of The First Affiliated Hospital of University of South China approved this study. Specimens were instantly stored in liquid nitrogen (−180°C) after biopsy.

### Cell Culture

The human NPC cell lines HNE1, HNE2, HNE3, CNE1, CNE2, 5-8F, and HK1 were obtained from Hunan Province Key Laboratory of Tumor Cellular & Molecular Pathology (University of South China, Hengyang, China). The cancer cells were cultured with RPMI Medium (Hyclone) with 10% FBS (Sigma) and antibiotics (100 units/mL of penicillin and 100 μg/mL streptomycin) at 37°C and 5% CO_2_.

### Total RNA Extraction

Total RNA was isolated from the frozen tissues by TRIzol (Invitrogen, USA) based on the manufacturer's instructions. The quantity of the isolated RNA of each sample was tested using ND-1000 spectrophotometer (NanoDrop/Termo, Wilmington, DE). The OD260/OD280 ratio was used as the RNA purity index. If the OD260/OD280 ratio ranges between 1.8 and 2.1, the purity of RNA is qualified, and the QC results are determined as “Pass.”

### Library Construction and circRNA Sequencing

CircRNA sequencing was performed by Aksomics Inc (Shanghai, China). A total quantity of 1~2 μg total RNA per sample was used to enrich circRNA using NEB Next® Poly(A) mRNA Magnetic Isolation Module (New England Biolabs, USA). Strand-specific CircRNA-seq libraries were constructed by KAPA Stranded RNA-Seq Library Prep Kit (KAPA, USA) with pretreated RNAs according to the manufacturer's protocol. The digested RNAs were fragmented into pieces and then used to synthesize first-strand and the second-strand cDNA. Next, the cDNA products were end-repaired, added a single “A” base, and Illumina sequencing adaptors were ligated onto the double-stranded cDNA. After libraries were purified and enriched by PCR, the quality of libraries was controlled by the Agilent 2100 Bioanalyzer (Agilent Technologies Inc, USA) using the Agilent DNA 1000 chip kit (Agilent Technologies Inc, USA). Finally, the double-stranded cDNA was denatured as single-stranded DNA and then sequenced for 150 cycles on an Illumina X-ten/NovaSeq system (Illumina, USA).

### Data Analysis and Differentially Expressed circRNA Identification

Raw sequencing data were under Quality Control (QC) and filtered to remove the joint sequence and too short clips. Then, the trimmed data was aligned to the reference genome (GRCh37/hg19) using STAR software (version 2.5.2b). Differential expression for circRNA-seq data were calculated using the edgeR package (version 3.20.9) in the statistical R program (version 3.5.0). Genes with a fold change ≥ 2.0 or fold change ≤ 0.5 and *p* < 0.05 between cases and controls were selected as differentially expressed circRNAs. For annotation, these differentially expressed circRNAs were blasted by the circBase (18), and those that cannot be annotated were defined as novel circRNAs. The sequencing data that cannot be aligned to reference genome directly were subjected to the subsequent circRNA analysis by recognition of the reverse splicing event using CIRCexplorer2 software (version 2.3.2) (19). These differentially expressed circRNAs were visualized with a circular view by CIRCOS visualization software (20).

### qRT-PCR Validation for the Expression of circRNA

Quantitative reverse-transcription polymerase chain reaction (qRT-PCR) was performed to validate the expression of circRNAs identified by sequencing. Six circRNAs annotated by circBase were selected, including the top three upregulated and three downregulated circRNAs. All the primers for candidate circRNAs are listed in **Table 2**, and these were purchased from Sangon Biotech (Shanghai, China). For qRT-PCR analysis, cDNA was synthesized from 1 μg of total RNA using the All-in-One First-Strand cDNA Synthesis kit (GeneCopoeia Inc, Santa Cruz, CA). The qRT-PCR analyses were performed by All-in-One qPCR Mix (GeneCopoeia Inc, USA) on ABI 7500HT qRT-PCR system (Applied Biosystems, Foster City, CA) as reactions: 95°C for 48 s, followed by 40 cycles of 95°C for 5 s and 62.5°C for 40 s. The reference gene was GAPDH, and the relative levels of gene expression were calculated using the 2^−ΔΔCt^ method.

### GO and KEGG Pathway Analysis of circRNAs

The potential functions of differentially expressed circRNAs and their parental genes were analyzed by DAVID (https://david.ncifcrf.gov/). GO enrichment analysis was performed, in terms of biological process (BP), cellular component (CC), and molecular function (MF), with Gene Ontology (http://geneontology.org/). The KEGG enrichment analysis revealed the significantly enriched pathways of the parental genes of differentially expressed circRNAs analyzed using KEGG (http://www.genome.jp/kegg/).

### CircRNA–miRNA–mRNA Interaction Prediction

CircRNAs as miRNAs sponges can indirectly interfere with the translation of the targeted mRNAs. We used miRanda software (version 3.3a) (21) (http://www.microrna.org/microrna/home.do) and RNAhybrid software (version 2.1.2) (22) (https://bibiserv.cebitec.uni-bielefeld.de/rnahybrid/) to predict the targeted miRNAs and mRNAs for hsa_circ_0002375 (circKITLG). The circRNA/miRNA/mRNA networks were visualized by Cytoscape software (version 3.7.0) (23) (https://cytoscape.org/).

### Transfection

Small interfering RNAs (siRNAs) targeting circKITLG backsplice junction sites and negative control (NC) oligonucleotides were purchased from RIBOBIO (Ribobio Co.,Ltd., Guangzhou, China). The siRNA sequences were si-circKITLG#1, 5′-GTACATTGACTTGGATTCTCA-3′; si-circKITLG#2, 5′- ACATTGACTTGGATTCTCACT-3′; si-NC, and 5′-TTCTCCGAACGTGTCACGT-3′. Cells were transfected with final concentrations of 50 nM of siRNAs using the Lipofectamine 2000 (Invitrogen, Carlsbad, CA, USA) according to the manufacturer's instructions.

### Cell Proliferation Assay

Cells were seeded into the 96-well plate (3 × 10^3^ cells/well). A cell proliferation assay was performed at the indicated time points using CCK-8 kit (CCK8, Beyotime, Nanjing, China) according to the manufacturer's instructions. The optical density (OD) at 450 nm was determined with a microplate reader (Thermo Fisher Scientific, Waltham, MA, USA).

### Colony Formation Assay

For colony formation assay, 24 h after transfection, cells were seeded into the 6-well plate (4 × 10^2^ cells/well). The culture medium was replaced every 3 days for 2 weeks. Then the colonies were fixed using methanol for 20 min and stained by crystal violet (Beyotime, Nanjing, China) for 15 min.

### Cell Migration and Invasion Assays

Cell migration ability was evaluated by wound healing assays. Cells were seeded into 6-well plates and scraped using 10 μl tips when cell confluence reached 90%. Cells were then cultured with serum-free medium for 48 h. The plates were photographed under a microscope at different time points. Cell invasion assays were conducted using Transwell chamber (Corning, NY, USA) coated with Matrigel (Corning, NY, USA). Cells suspended with serum-free medium were seeded into the upper chamber (2 × 10^4^ cells/well), and the culture medium with 10% FBS was placed into the lower chamber. After 48 h, the cells on the upper surfaces of the Transwell chamber were removed with cotton swabs, and the cells on the lower surfaces were fixed with paraformaldehyde and stained by crystal violet. The stained cells were photographed and counted under a microscope.

### Statistical Analysis

The statistical analyses were performed by SPSS18 software (SPSS Inc., IL, USA). The results were presented as the mean ± SD. For analysis, we used Student's *t*-test and One-way ANOVA between groups, and *P* < 0.05 was considered to be statistically significant. More specifically, ^*^P < 0.05; ^**^P < 0.01; and ^***^P < 0.001.

## Results

### CircRNA Expression Profile in NPC

**Figure 1:**
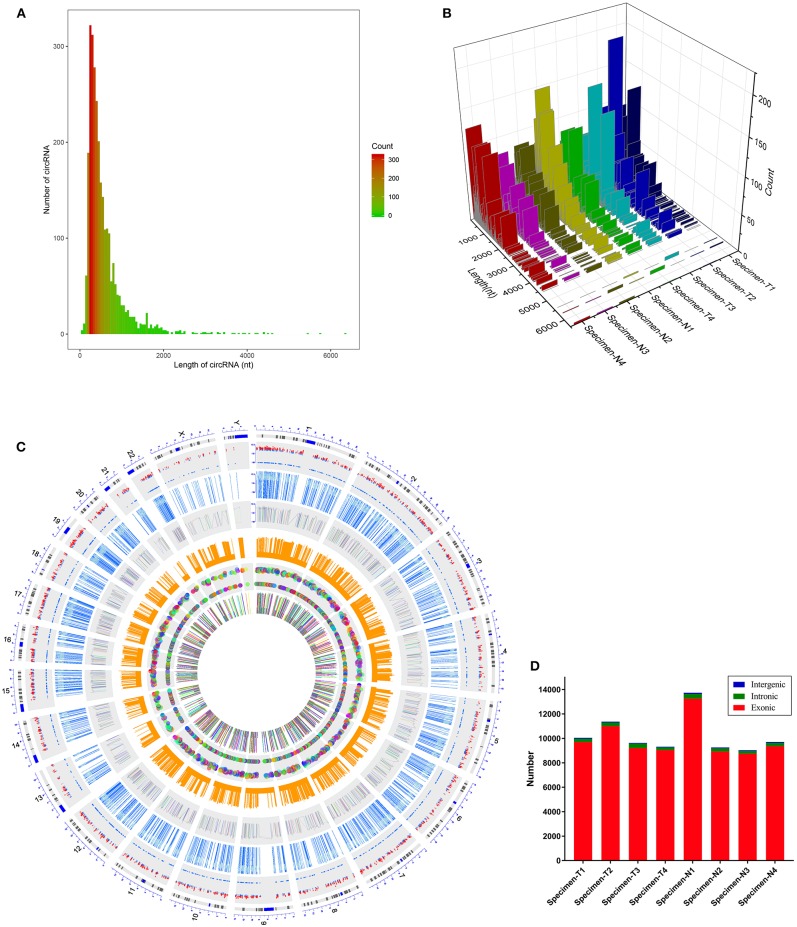
Characteristics of the identified circRNAs in nasopharyngeal carcinoma and healthy controls. **(A,B)** Length distribution of the identified circRNAs. x-axis, the length of the detected circRNAs; y-axis, the abundance of circRNAs classified by different lengths. **(C)** Distribution of the candidate circRNAs on chromosomes. **(D)** Genomic origin of the identified circRNAs.

We first analyzed the circRNA expression profile of four matched tissues acquired from NPC and nasopharyngitis patients by high-throughput deep sequencing. More than 12 gigabytes (Gb) of sequenced data of each specimen were aligned to the reference genome (GRCh37/hg19) using STAR software (version 2.5.2b). A total of 2,855 circRNAs were identified in these samples. Of these, 192 circRNAs were previously unknown. The lengths of candidate circRNAs were mostly <2000 nucleotides (nt) (Figures 1A,B). These candidate circRNAs were annotated using the RefSeq database. The candidate circRNAs were distributed on all chromosomes, including sex chromosomes X and Y (Figure 1C). Among these candidate circRNAs' host genes, 96.5% originated from exonic regions, and the rest lay on intronic and unannotated regions (Figure 1D).

**Figure 2:**
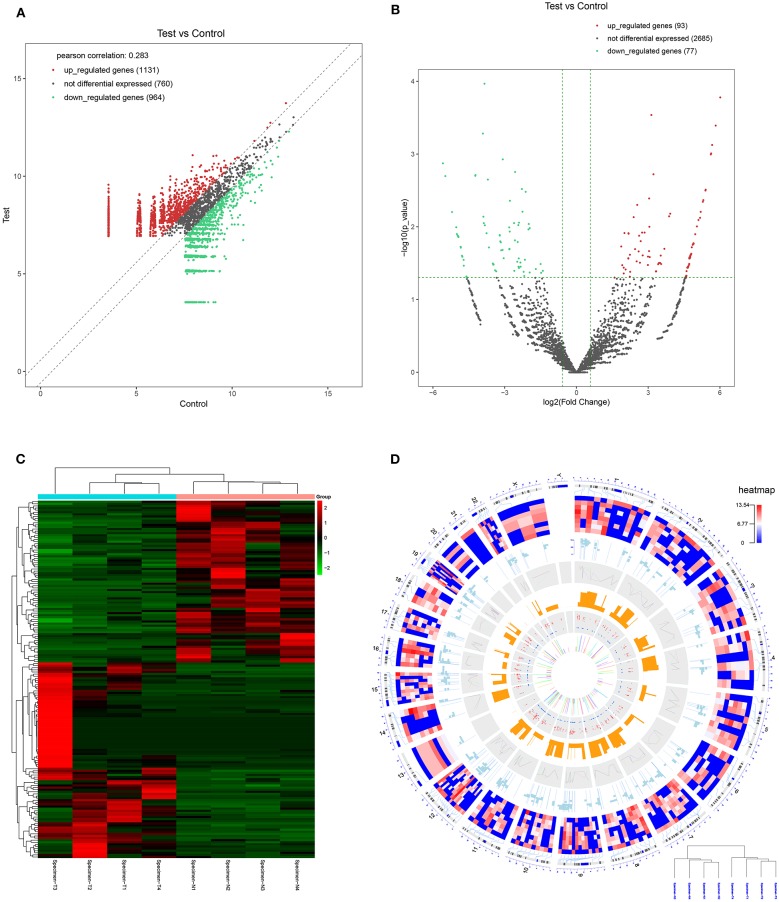
CircRNA expression profile in nasopharyngeal carcinoma compared to healthy controls. **(A)** The expression of circRNAs in the four pairs of nasopharyngeal carcinoma tissues and healthy controls through the high-throughput sequencing. **(B)** Volcano plots of the differentially expressed circRNAs. Horizontal dotted line: *P* = 0.05 (–log10 scaled); red points: upregulated circRNAs with statistical significance; green points: downregulated circRNAs with statistical significance. **(C)** Heat map and hierarchical cluster analysis of all target circRNAs. Red strip: high relative expression; blue strip: low relative expression. **(D)** Heat map of differentially expressed circRNAs on human chromosomes.

CircRNA expression profile was used to evaluate the variations between the NPC tissue specimens group and four matched normal tissue specimens group using the scatter plot (Figure 2A). Differentially expressed circRNAs with a statistical significance between the two groups were identified with fold change ≥ 2.0 or fold change ≤0.5 and *p* < 0.05. A total of 170 circRNAs were significantly differentially expressed, including 93 remarkably upregulated circRNAs and 77 significantly downregulated circRNAs visualized by volcano plots (Figure 2B) and a cluster heatmap (Figure 2C). The top 20 differentially expressed circRNAs are presented (Table 1). The differentially expressed circRNAs were distributed by heatmap on human chromosomes (Figure 2D).

**Table 1:** Top 20 differentially expressed circRNAs in the nasopharyngeal carcinoma.

**circRNA_ID**	**Chr**	**Locus**	**Strand**	**Gene_Name**	**Length**	**log2FC**	***p*-value**
hsa_circ_0002375	chr12	88898935–88939642	-	KITLG	844	6.0146682	0.00017
hsa_circ_0111974	chr1	216824314–216850833	-	ESRRG	533	5.8223723	0.00040
chr10:46121398-46135411:-	chr10	46121398–46135411	-	ZFAND4	1303	5.6740423	0.00075
hsa_circ_0081534	chr7	100417178–100417918	-	EPHB4	489	5.6344749	0.00098
hsa_circ_0025767	chr12	29904598–29911710	-	TMTC1	458	5.6178356	0.00101
hsa_circ_0088018	chr9	114190321–114195652	-	KIAA0368	342	5.4035426	0.00309
hsa_circ_0135761	chr8	132952745–132958880	+	EFR3A	356	5.3922431	0.00316
hsa_circ_0094943	chr11	110007387–110034104	+	ZC3H12C	1234	5.3060947	0.00430
hsa_circ_0079557	chr7	22306582–22357656	-	RAPGEF5	565	5.2869091	0.00453
hsa_circ_0066568	chr3	78763546–78767033	-	ROBO1	388	5.1949179	0.00622
hsa_circ_0007439	chr2	29006772–29011675	+	PPP1CB	224	−5.5835715	0.00134
chr19:6697354-6697805:-	chr19	6697354–6697805	-	C3	356	−5.4836046	0.00201
hsa_circ_0000345	chr11	77409531–77413540	-	RSF1	1982	−5.2024664	0.00624
chr19:42621401-42664544:-	chr19	42621401–42664544	-	POU2F2	336	−5.0259181	0.01015
hsa_circ_0138314	chr9	14672827–14680160	-	ZDHHC21	428	−5.0014845	0.01256
hsa_circ_0105201	chr16	22162015–22163955	+	VWA3A	276	−4.9682426	0.01319
chr9:100092435-100093049:+	chr9	100092435–100093049	+	AL512590.3	486	−4.9654478	0.01325
hsa_circ_0001913	chrX	19701940–19713859	-	SH3KBP1	336	−4.9523179	0.01341
hsa_circ_0004315	chr16	74491771–74493687	-	GLG1	229	−4.9392393	0.01356
hsa_circ_0025967	chr12	46319924–46322642	-	SCAF11	2718	−4.932998	0.01380

### Validation of Differentially Expressed circRNAs by qRT-PCR

To validate the RNA-seq results, the top three upregulated and three downregulated circRNAs annotated by circBase were selected for validation by qRT-PCR with outward-facing primers (Table 2) blasted to the circRNA transcripts.

**Table 2:** A list of primers (F, forward and R, reverse) used in this study.

**circRNA**	**Primer sequence**
hsa_circ_0002375	F:5′- GTTGCAAGAGAAAGAGAGAGAGTT-3′
	R:5′- CGATTCCTGCAGATCCCTTC-3′
hsa_circ_0111974	F:5′- TCACAAAGCGCAGACGTAAA-3′
	R:5′- ATGAAGGACGAACAGCTGGAA-3′
hsa_circ_0081534	F:5′- GAACGGGGTATCCTCCTTAGC-3′
	R:5′- TTAGAGTGGCTATTGGCTGGG-3′
hsa_circ_0007439	F:5′- GGGAGAAAATGATCGTGGTGTT-3′
	R:5′- AGATTGCAGGTCTGGTGACAA-3′
hsa_circ_0000345	F:5′- GTGAAGAAGCCATCCTAGCAGA-3′
	R:5′- AGAACATTGGCTGTAGAACGGT-3′
hsa_circ_0138314	F:5′- GTCTGGTTGCCTTAGTGAGGG-3′
	R:5′- TCCCACCAAATGGATCTCTCTTC-3′
GAPDH	F:5′- CATGAGAAGTATGACAACAGCCT-3′
	R:5′-AGTCCTTCCACGATACCAAAGT-3′

*F, forward; R, reverse*.

**Figure 3:**
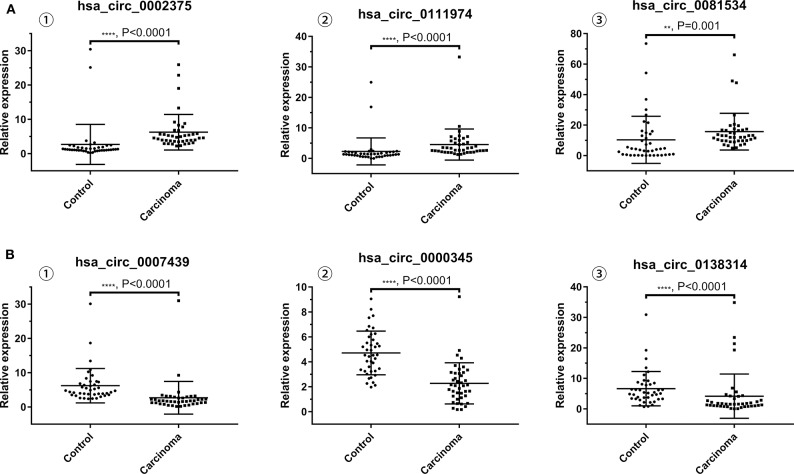
Validation of the expression level of six selected circRNAs. **(A)** The levels of the top three upregulated circRNAs (hsa_circ_0002375, hsa_circ_0111974, and hsa_circ_0081534) in NPC tissues are significantly higher than normal tissues. **(B)** The levels of the top three downregulated circRNAs (hsa_circ_0007439, hsa_circ_0000345, and hsa_circ_0138314) in NPC tissues are significantly lower than normal tissues.

The results of qRT-PCR revealed that the expression levels of hsa_circ_0002375 (circKITLG) (*P* < 0.0001), hsa_circ_0111974 (circESRRG) (*P* < 0.001), and hsa_circ_0081534 (circEPHB4) (*P* = 0.001) were significantly upregulated in the NPC specimens (Figure 3A), and the expression levels of hsa_circ_0007439 (circPPP1CB) (*P* < 0.0001), hsa_circ_0000345 (circRSF1) (*P* < 0.001), and hsa_circ_0138314 (circZDHHC21) (*P* < 0.0001) were significantly downregulated in the NPC specimens (Figure 3B).

### GO and KEGG Pathway Analyses of Differentially Expressed circRNAs

**Figure 4:**
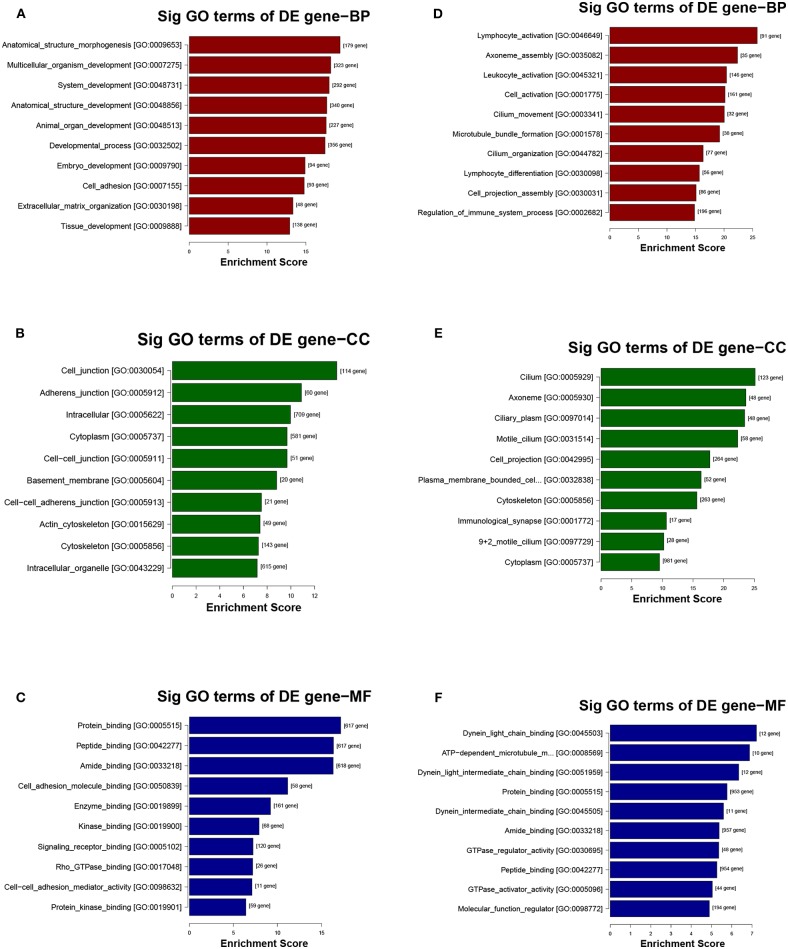
GO analysis of the parental genes of the differentially expressed circRNAs, includes the following categories: biological process (BP), cellular component (CC) and molecular function (MF). **(A–C)** GO analysis corresponds with the upregulated circRNAs. **(D–F)** GO analysis corresponds with the downregulated circRNAs.

**Figure 5:**
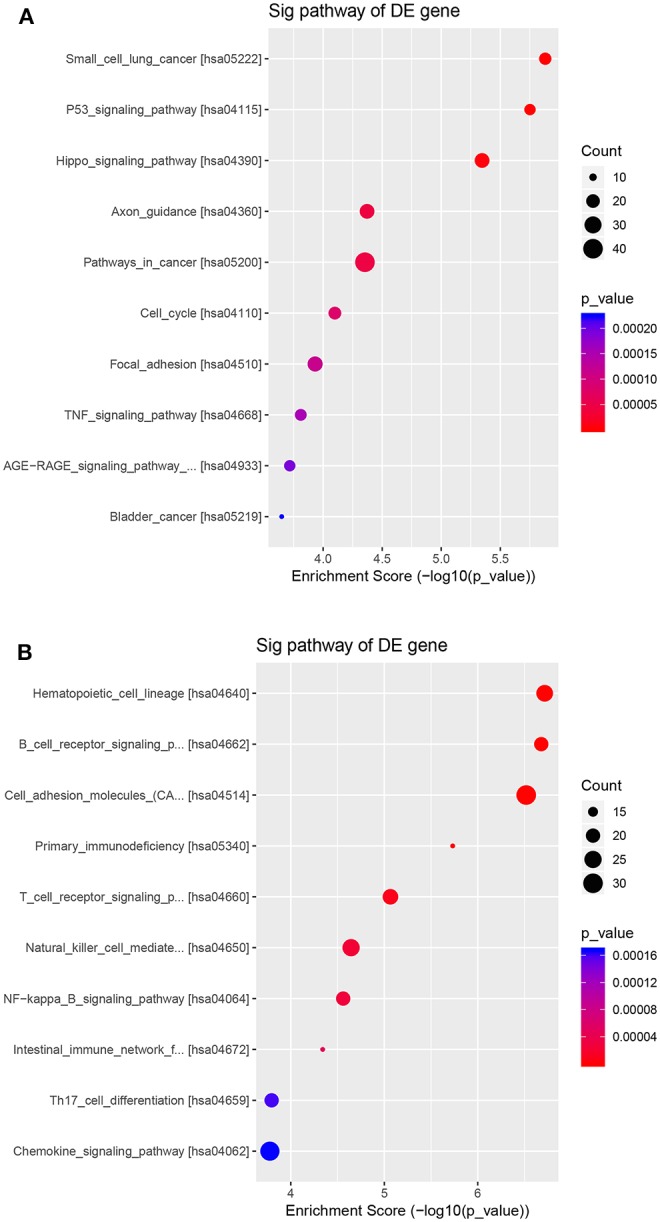
KEGG pathway analyses of the parental genes of the differentially expressed circRNAs. The pathways correspond with **(A)** the upregulated circRNAs and **(B)** the downregulated circRNAs. The results are shown in a scatter plot. Enrichment factor refers to the ratio of the number of genes located in the pathway entry and the total number of genes in the pathway entry.

The potential function and connection of the differentially expressed circRNAs and their parental genes were predicted using GO and KEGG analyses. The top 10 enrichment GO terms for differentially expressed circRNAs are shown. The most significant enriched GO terms in the biological process were related to the anatomical structure morphogenesis process (GO:0009653) and lymphocyte activation process (GO:0046649) (Figures 4A,D); the most significant enriched GO terms in cellular component were related to the cell junction (GO:0030054) and cilium process (GO:0005929) (Figures 4B,E); and the most significant enriched GO terms in molecular function were related to the protein binding (GO:0005515) and dynein light chain binding process (GO:0045503) (Figures 4C,F). The top 10 enriched pathways of KEGG pathway enrichment analysis are displayed in an enriched scatter diagram (Figures 5A,B). These results showed that the differentially expressed genes might be associated with tumor signaling pathways and immune signaling pathways.

### Prediction of the circRNA–miRNA–mRNA Interaction Network

**Figure 6:**
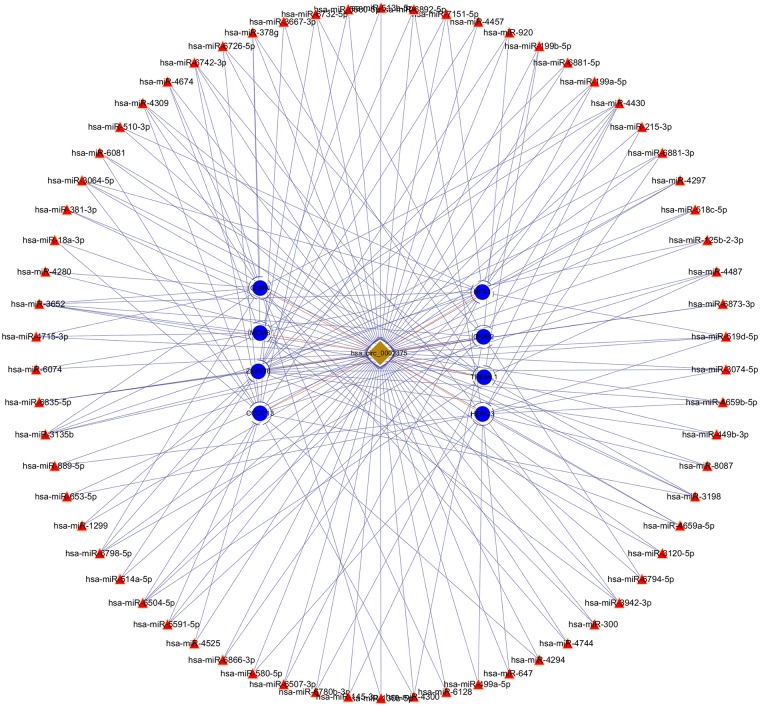
CircRNA-miRNA-mRNA network analysis of the top upregulated circRNA hsa_circ_0002375 (circKITLG).

The top upregulated circRNA hsa_circRNA_002375 (circKITLG), validated by qRT-PCR, was selected for analyzing the network between circRNAs and miRNAs by miRanda and RNAhybrid software. Only the circRNA and miRNA interactions predicted using both tools were considered. The potential functional network of circKITLG were presented (Figure 6).

### Silencing of circKITLG Inhibited NPC Cell Proliferation, Migration, and Invasion *in vitro*

**Figure 7:**
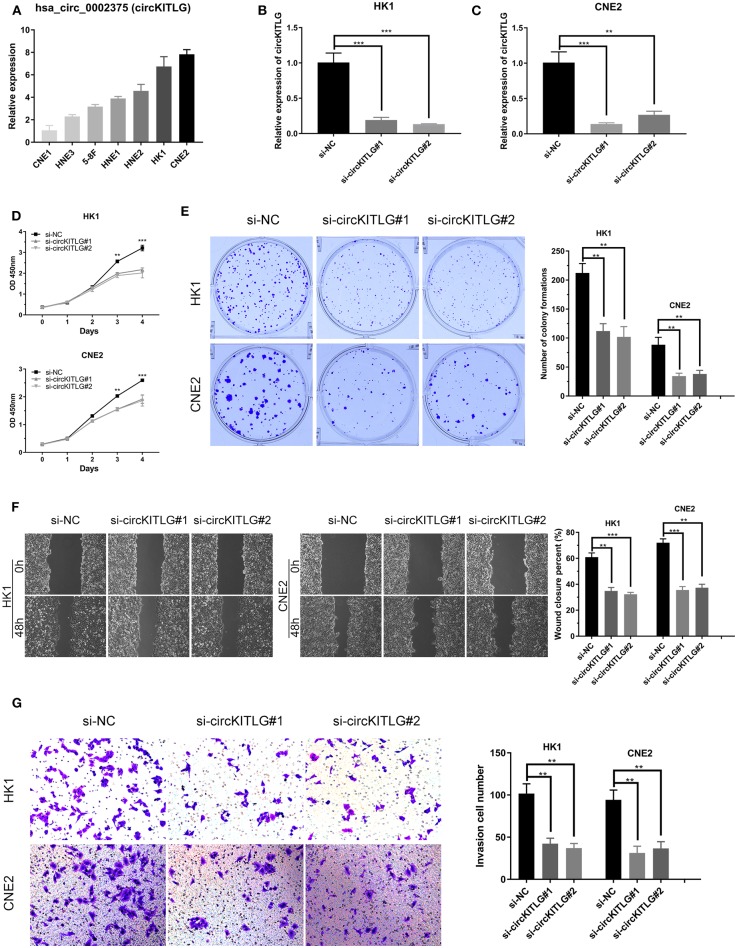
Silencing of hsa_circ_0002375 (circKITLG) inhibited NPC cell proliferation, migration, and invasion. **(A,B)** Relative expression of circKITLG in 7 NPC cell lines was examined by qRT-PCR. **(C)** Results of qRT-PCR for circKITLG in HK1 and CNE2 cells treated with circKITLG siRNAs. **(D)** CCK-8 assay was performed to evaluate the growth of NPC cells after treatment with circKITLG siRNAs. **(E)** Colony formation assay was performed after transfected with NC or circKITLG siRNA. **(F,G)** Migration and invasion of NPC cells transfected with NC or circKITLG siRNA. **p < 0.01 and ***p < 0.001.

As shown in Table 1 and Figure 3A, circKITLG was the top upregulated circRNA and validated by qRT-PCR. Hence, to further explore the biological functional roles of circRNAs in NPC *in vitro*, we selected circKITLG as a candidate circRNA for further investigation. The expression of circKITLG in seven NPC cell lines was measured by qRT-PCR, and the results demonstrated that circKITLG expression level was higher in HK1 and CNE2 cells than others (Figure 7A). Therefore, HK1 and CNE2 cell lines were chosen for silencing of circKITLG (Figures 7B,C). Cell proliferation was measured by the CCK-8 assay, and knockdown of circKITLG significantly attenuated cell proliferation in both HK1 and CNE2 cells (Figure 7D). The colony formation abilities of NPC cells were also markedly inhibited by silencing circKITLG (Figure 7E). Moreover, in the wound healing assays and transwell invasion assays, silencing of circKITLG significantly inhibited the migration and invasion abilities of HK1 and CNE2 cells (Figures 7F,G). Therefore, the above results demonstrated that silencing of circKITLG could inhibit the progression of NPC cells.

## Discussion

CircRNAs, as a burgeoning sort of non-coding RNA, could function as miRNA sponges, which can regulate the expression of parental genes (24, 25). A significant number of studies have revealed that circRNAs are implicated in various human diseases, including carcinomas (26). However, no NPC-associated circRNA has been identified by high-throughput sequencing. In this study, we presented a leading and primary circRNA expression profile in NPC using high-throughput sequencing.

In our research, a total of 93 upregulated circRNAs and 77 downregulated circRNAs were identified. Hsa_circ_0002375, hsa_circ_0111974, and hsa_circ_0081534 were the significantly upregulated circRNAs, and hsa_circ_0007439, hsa_circ_0000345, and hsa_circ_0138314 were the significantly downregulated circRNAs confirmed by qRT-PCR. After validation using qRT-PCR, six selected circRNAs were consistent with the RNA-seq data. These differentially expressed circRNAs may be used as biomarkers and therapeutic targets of NPC, while the exact roles of circRNAs requires further investigation.

CircRNAs were encoded from exons and/or introns of their parental genes (27). Our data showed that most of the circRNAs are from exons. Exonic circRNA is generated by the back-slicing process, an out-of-order arrangement of exons (28–30). Hsa_circ_0002375, hsa_circ_0111974, and hsa_circ_0081534 are spliced from KIT ligand (KITLG), estrogen related receptor gamma (ESRRG), and EPH receptor B4 (EPHB4), respectively, which play an essential role in cancer proliferation, metastasis and apoptosis. KITLG is the ligand of the tyrosine-kinase receptor, which is demonstrated as a novel target of miR-34c that inhibited the growth and invasion of colorectal cancer cells (31). ESRRG is a member of the estrogen receptor-related receptor (ESRR) family, which has been identified as a tumor suppressor gene in several cancers (32–36). EPHB4 is one of the EphB subfamily, the largest of receptor tyrosine kinases, which is known to facilitate vascularization in multiple carcinomas and is upregulated in various cancers, including upper aerodigestive cancers (37–40). Based on this, we think that circRNA may participate in development and prognosis of NPC.

According to the GO and KEGG pathway analyses, we explored the biological functions and potential mechanisms of circRNAs in NPC. We found that cell junction and cilium process affect the development of NPC. Moreover, the P53 and Hippo pathway has been shown to be related to the development and prognosis of NPC. CircRNAs, acting as miRNAs sponge, regulate the miRNA to impact cancer development and progress. Therefore, in this study, we predict a relationship between the circRNA and microRNA by *in silico* analyses. For example, the top upregulated circRNA hsa_circ_0002375 (circKITLG) potentially binds miR-3198. Kanzaki H et al. provided experimental evidence for the role of miR-3198 in in periodontal ligament cells through downregulates OPG expression in response to mechanical stress (41). *In vitro* experiments showed that knockdown of circKITLG could inhibit NPC cell proliferation, migration, and invasion. Each of these proves that circRNAs play an important role in NPC. To confirm whether these circRNAs are involved in the development of NPC, further functional and mechanistic studies and a larger cohort of patients are required.

In summary, we found that circRNAs were significantly differentially expressed in NPC compared with normal tissues in this study. CircRNAs play a key role in the development and progress of NPC and regulates cancer-related pathways. This study will help researchers to elucidate the mechanism of NPC tumorigenesis and progression and provide new clinical diagnostic markers and therapeutic targets.

## Data Availability Statement

The data in this article can be found in the GEO database with the link: https://www.ncbi.nlm.nih.gov/geo/query/acc.cgi?acc=GSE143797.

## Ethics Statement

The studies involving human participants were reviewed and approved by The Medical Ethics Committee of The First Affiliated Hospital of University of South China. The patients/participants provided their written informed consent to participate in this study. Written informed consent was obtained from the individual(s) for the publication of any potentially identifiable images or data included in this article.

## Author Contributions

ZL designed this project. JY, YG, QJ, LL, SL, QZ, and FH performed the experiments.

## Conflict of Interest

The authors declare that the research was conducted in the absence of any commercial or financial relationships that could be construed as a potential conflict of interest.
